# 180th Anniversary of Ludwig Boltzmann

**DOI:** 10.3390/e27040394

**Published:** 2025-04-07

**Authors:** Antonio M. Scarfone, Sérgio Luiz E. F. da Silva

**Affiliations:** 1 Istituto dei Sistemi Complessi—Consiglio Nazionale delle Ricerche (ISC-CNR), c/o Dipartimento di Scienza Applicata e Tecnologia del Politecnico di Torino, 10129 Torino, Italy; antoniomaria.scarfone@cnr.it; 2 Laboratory of Parallel Architectures for Signal Processing, Federal University of Rio Grande do Norte, Natal 59078-970, Brazil

Everybody who has devoted themselves in some way to the study of the scientific disciplines knows the giant contribution that Ludwig Boltzmann made to theoretical physics. Born in Vienna on 20 February 1844 into a world on the cusp of scientific revolution, Boltzmann showed an aptitude for mathematics and physics from a young age. Influenced by prominent scientists such as Christian Doppler and Josef Stefan, who followed him from his early studies at the University of Vienna, he completed his doctoral thesis in 1866 on the behavior of gases, a topic that would become central to most of his future works and which would mark him in the following century as one of the most important figures in the history of physics, alongside contemporaries such as James Clerk Maxwell and Josiah Willard Gibbs, establishing foundational principles that continue to shape the field of statistical physics to this day.

By the age of 25, he had already been appointed as a full professor of mathematical physics at the University of Graz, Austria. Widely regarded as one of Austria’s greatest scientists, Boltzmann received invitations to occupy prestigious chairs in theoretical physics at leading academic centers, most notably in Berlin, Germany. In addition, he was awarded numerous honorary titles, reflecting the high esteem in which he was held by the scientific community.

His profound interest in thermodynamics shaped Boltzmann’s early academic career, beginning the development of the kinetic theory of gas by introducing the idea of monads in a modern key and by highlighting the necessity of employing statistical methods in physics [1]. In 1872, by the age of only 28, he established the groundwork for contemporary statistical mechanics with a pair of influential papers: “Weitere studien über das wärmegleichgewicht unter gasmolekülen” (Further Studies on the Thermal Equilibrium of Gas Molecules) [2] and “Über die beziehung eines allgemeinen mechanischen satzes zum zweiten hauptsatz der wärmetheorie” (On the relationship of a general mechanical theorem to the second law of thermodynamics) [3]. In these papers, Ludwig Boltzmann introduced groundbreaking concepts such as the transport equation that bears his name and the statistical interpretation of entropy. His incorporation of probabilistic reasoning into physics revolutionized our comprehension of thermodynamics, entropy, and transport theory and provided insights into the second law of thermodynamics, which is central to modern physics, facilitating subsequent scientific advancements that continue to shape the various disciplines.

Boltzmann, by studying the approach to equilibrium in a system, introduces following the kinetic equation:

(1) $$\frac{\partial }{\partial t}f\left(v,\;t\right)+a\cdot \nabla_{v}f\left(v,\;t\right)=C\left[f\left(v,\;t\right)\right]\;,$$

today named the Boltzmann equation, for a single-particle probability distribution function f(v,t), where C[f], the collisional term, accounts for the binary interactions among the monads of the system. In this way, he was able to introduce the so-called H-functional,

(2) H[f]=∫f(v,t)lnf(v,t)dv

which, together with the relation dH/dt≤0, constitutes his celebrated H-theorem.

Several years later, Josiah Willard Gibbs [4], a pioneer in the study of statistical mechanics, introduced the well-known expression

(3) $$S\left[p\right]=-k_{B}\int p\left(x,\;v,\;t\right)\;\ln\left(p\left(x,\;v,\;t\right)\right)\;dx\;dv\;,$$

which shows an evident similarity with the H-functional (2), where p(x,v,t) is the probability distribution function of the system in the phase space. The dimensional constant $k_{B}$ has since been baptized in honor of Ludwing as the Boltzmann constant. This relation (3) is now known as Boltzmann–Gibbs entropy. It is used in statistical mechanics to measure how disordered or random a system with many parts is on a microscopic level. The legitimacy of this last statement finds validity in the following expression:

(4) $$S=k_{B}\;\ln\left(W\right)\;,$$

written in this form by Max Planck [5] during his studies on black-body radiation. Although Boltzmann never explicitly wrote this last relation, it is nowadays known as the Boltzmann–Planck formula of entropy. Epigraphed on the Boltzmann tomb as a testimony to his enormous contribution to this field, it is a pillar in the field of thermostatistics.

However, Boltzmann’s ideas, probably due to their extraordinary innovation, were not largely accepted and recognized as such initially by the wider scientific community. These have given rise to several controversial discussions with prominent personalities, such as Ernst Mach, and have also given rise to the birth of logical paradoxes like Maxwell’s devil, the Poincare recurrence, and the Loschmidt paradox, to cite the most well known, which, in some way, exist in contrast to the prediction made by the H-theorem, and which have only recently found partial explanations.

Furthermore, Boltzmann’s contributions to the understanding of heat, energy, and the behavior of gases in terms of statistical laws have surely revolutionized the way scientists today approach the behavior of matter. The principles he introduced have profoundly influenced not only physics but also fields such as biology, engineering, and even economics, where statistical approaches play a fundamental role in the understanding of many complex systems. A complete compilation of Ludwig Boltzmann’s published works, including all his articles originally published in journals, can be found in ref. [6].

This Special Issue celebrates the 180th anniversary of Ludwig Boltzmann’s birth by bringing together high-quality reviews and original research articles that examine the enduring influence of Boltzmann’s concepts on statistical physics and related fields. Our aim is not only to acknowledge Boltzmann’s foundational contributions but also to explore how his pioneering ideas continue to inspire advancements at the forefront of contemporary science and technology. The breadth of research presented here demonstrates the ongoing relevance of statistical mechanics in diverse domains.

The articles featured in this Special Issue illustrate the far-reaching impact of Boltzmann’s work. Tamburrini, Davis, and Moya (2023) [7] introduce the Ehrenfest procedure as an efficient alternative to probability density functions for macroscopic properties in systems with multiple degrees of freedom, such as plasmas. This approach, rooted in the conjugate variable theorem and fluctuation–dissipation theorem, offers a computationally efficient method for studying adiabatic invariants in magnetized plasmas. Their study validates theoretical predictions through test particle simulations, demonstrating the utility of statistical mechanics in understanding dynamical systems far from equilibrium.

Kais (2024) [8] investigates quantum phase transitions in finite systems, challenging the conventional view that true criticality only emerges in the thermodynamic limit. Traditionally, finite-size scaling methods have been employed to extrapolate results to larger systems, but recent advances in ultra-cold atomic and molecular systems have raised new questions about the nature of phase transitions in genuinely finite systems. By developing a finite-size scaling approach tailored for such cases, this study examines quantum critical parameters in the context of chemical processes at ultra-cold temperatures. The recent observation of a quantum phase transition in a single trapped ^171^Yb^+^ ion suggests that these transitions may play a fundamental role in chemical bond formation and dissociation, offering fresh perspectives on quantum chemistry.

Expanding on the statistical framework for microscopic systems, Alvarez-Estrada (2024) [9] extends a previous analysis of the quantum Wigner function and non-equilibrium equations for a microscopic particle in one spatial dimension. By deriving new solutions for the hierarchy equations and linking them to a Smoluchowski equation, this study develops a model describing molecular chain growth through atomic binding and catalyst activation in three-dimensional space. This work provides valuable insights into stochastic dynamics at the microscopic scale.

Rondoni and Di Florio (2024) [10] contribute to the fundamental understanding of the Boltzmann equation by analyzing conditions under which probability behaves analogously to mass. They emphasize that probability is the only suitable mathematical tool for describing small systems that deviate from the classical Boltzmann equation. Their study underscores the continued relevance of Boltzmann’s insights in interpreting statistical mechanics applications.

From a conceptual perspective, Gimbel (2024) [11] explores the broader implications of Boltzmann’s statistical approach to entropy. This study highlights how his 1877 formulation introduced quantization, reformulated entropy, and challenged deterministic interpretations of the laws of nature. Boltzmann’s statistical perspective on the Second Law of Thermodynamics marked a paradigm shift, aligning with other scientific developments of the time, such as Darwin’s theory of evolution and Hutton’s geological theories. This paper also examines potential influences between Boltzmann and Darwin, noting Boltzmann’s frequent references to evolutionary theory in his later writings.

Edrisi, Patwa, and Morales Escalante (2024) [12] examine Wehrl entropy for harmonic potential benchmark problems, deriving a steady-state analytical formula. However, their findings indicate a lack of theoretical results on absolute entropy monotonicity in the presence of friction. By employing a stochastic Monte Carlo numerical solver, this study provides numerical evidence suggesting the possibility of monotonic behavior, shedding light on entropy evolution in non-equilibrium systems.

In the domain of granular suspensions, Gómez González and Garzó (2024) [13] investigate non-Newtonian transport properties using inelastic Maxwell models and BGK-type kinetic approaches. Their study explicitly accounts for collisions between granular particles and a molecular gas, contrasting with traditional coarse-grained models. By employing both inelastic Maxwell models (IMM) and BGK-type kinetic models, they provide exact transport property computations and detailed velocity moment analyses. Notably, their findings highlight the phenomenon of discontinuous shear thickening (DST), demonstrating its dependence on mass ratios and other parameters.

Hunt, Sahimi, and Newman (2024) [14] apply percolation theory and ecological optimality principles to refine predictions of plant species richness based on net primary productivity. Their approach enhances species-energy theory by offering a more precise expression for primary productivity as a function of precipitation and potential evapotranspiration, improving our understanding of biodiversity patterns and non-climatic influences on ecosystem dynamics.

Addressing the dynamics of complex networks, van Elteren, Quax, and Sloot (2024) [15] identify two critical roles that nodes play in catalyzing abrupt transitions. Initiator nodes act as amplifiers of short-lived fluctuations, destabilizing neighboring nodes, while stabilizer nodes encode long-term memory, counteracting cascading effects. This study introduces a new framework for understanding and controlling endogenous metastability in networked systems, with implications for fields ranging from epidemiology to financial systems.

In the realm of materials science, Svyetlichnyy (2025) [16] simulates the powder bed fusion (PBF) process for functionally graded materials (FGM) fabrication using a combination of unity-based deposition models and lattice Boltzmann method (LBM) simulations. By introducing a diffusion model for material mixtures, specifically AISI 316L austenitic steel and 18Ni maraging 300 martensitic steel, this study demonstrates consistency with experimental data. The proposed approach offers a powerful tool for simulating, studying, and optimizing FGM production in PBF processes, reinforcing the practical applications of Boltzmann’s methods in modern engineering.

Finally, Boguñá and Serrano (2025) [17] present a statistical mechanics framework for directed networks, conceptualizing them as ensembles of interacting fermions. Their approach systematically models network structures and dynamics, introducing novel perspectives and analytical tools for studying directed networks. By addressing reciprocity and other network characteristics, their work provides a principled method for investigating influence propagation and connectivity in complex systems.

We are honored to present this Special Issue as a tribute to Ludwig Boltzmann’s scientific vision. The breadth and depth of the research included here reaffirm the foundational role of statistical mechanics, and we hope the articles included herein will inspire future investigations into the challenges and opportunities that lie ahead.
